# Supplementary material for: Microglia Mitigate Neuronal Activation in a Zebrafish Model of Dravet Syndrome
Source: Cells. 2024 Apr 15;13(8):684. doi: 10.3390/cells13080684 (PMC11049242; doi:10.3390/cells13080684)
Supplement: Supplementary file 1 [file cells-13-00684-s001.zip › cells-2926156 supp info.pdf]

Supplementary video 1: 3D reconstruction of a representative microglia from a control brain's larvae (**Figure 1A**). 3D images were generated using Imaris software (Biplane Inc., Version 9.5.0). (Link: https://urlz.fr/iPxZ).

Supplementary video 2: 3D reconstruction of a representative microglia from a *scn1Lab*-KD brain's larvae (**Figure 1B**). 3D images were generated using Imaris software (Biplane Inc., Version 9.5.0). (Link: https://urlz.fr/iPxP).

Supplementary video 3: Time-lapse of 4 dpf control larvae dorsal view showing microglial displacement and processes dynamic (**Figure 2A**). Interval between frames: 39 s. Video played at x6. Microglial tracking displacement was performed using Imaris software (Biplane Inc., Version 9.5.0). (Link: https://urlz.fr/iOIz).

Supplementary video 4: Time-lapse of 4 dpf *scn1Lab* larvae dorsal view showing microglial displacement and processes dynamic (**Figure 2B**). Interval between frames: 39 s. Video played at x6. Microglial tracking displacement was performed using Imaris software (Biplane Inc., Version 9.5.0). (Link: https://urlz.fr/iOIA).

Supplementary Video 5: Representative time-lapse recording of calcium activity in the optic tectum of control larvae (**Figure 4B –** control with microglia). Interval between frames: 200 ms. Video played at x5.

Supplementary Video 6: Representative time-lapse recording of calcium activity in the optic tectum of *scn1Lab*-KD larvae (**Figure 4B –** *scn1Lab*-KD with microglia). Interval between frames: 200 ms. Video played at x5.

Supplementary Video 7: Representative time-lapse recording of calcium activity in the optic tectum of control larvae without microglia (**Figure 4B –** control w/o microglia). Interval between frames: 200 ms. Video played at x5.

Supplementary Video 8: Representative time-lapse recording of calcium activity in the optic tectum of *scn1Lab*-KD larvae without microglia (**Figure 4B –** *scn1Lab*-KD w/o microglia). Interval between frames: 200 ms. Video played at x5.

Supplementary table

il-1β	Forward	5′-CTT AAC CAG CTC TGA AAT GAT G-3′
	Reverse	5′-TGT CGC ATC TGT AGC TCA TTG-3′
il-8	Forward	5′-TGA CCA TCA TTG AAG GAA TGA G-3′
	Reverse	5′-CAT CAA GGT GGC AAT GAT CTC-3′
tnf-α	Forward	5′-TCA CGC TCC ATA AGA CCC AG-3′
	Reverse	5′-GAT GTG CAA AGA CAC CTG GC-3′
il-4	Forward	5′-GAG ACA GGA CAC TAC TCT AAG-3′

	Reverse	5′-GTT TCC AGT CCC GGT ATA TG-3′
il-10	Forward	5′-AAC GAG ATC CTG CAT TTC TAC-3′
	Reverse	5′-CCT CTT GCA TTT CAC CAT AT-3′
tgf-β3	Forward	5′-AAA ACG CCA GCA ACC TGT TC-3′
	Reverse	5′-CCT CAA CGT CCA TCC CTC TG-3′
